# Supplementary material for: Cloud BioLinux: pre-configured and on-demand bioinformatics computing for the genomics community
Source: BMC Bioinformatics. 2012 Mar 19;13:42. doi: 10.1186/1471-2105-13-42 (PMC3372431; doi:10.1186/1471-2105-13-42)
Supplement: Additional file 1 — Supplementary 1 Cloud BioLinux software documentation in the form of a mini, self-contained website. Users need to download and uncompress the .zip file, and open through a web browser the "index.html" file available on the main directory. (ZIP 1823 kb). [file 1471-2105-13-42-S1.ZIP › Cloud-BioLinux-Package-Documentation/docs/mcx.html]

Bio-Linux Software Documentation Pages

Back to search form

## mcx

|  |  |
| --- | --- |
| Name | mcx |
| Description | **mcx** is a stack language interpreter for interaction with the mcl libraries (a part of the MCL suite.  **mcx** can be used both from the command line and interactively, and supports a rich set of operations such as transposition, scaling, column scaling, multiplication, Hadamard powers and products, et cetera.  MCL is the Amsterdam implementation of the Markov Cluster Algorithm, written by Stijn van Dongen.  If you use this software in writing scientific papers, include proper citations (as found in the MCL manual page) and proper attributions to mcl's home on http://micans.org/mcl/ (or the Debian package on http://packages.debian.org/unstable/math/mcl.html) and its author, Stijn van Dongen.  In case you use the TribeMCL module, include proper citations to the TribeMCL copyright holder EMBL-EBI and its author Anton Enright. |
| Homepage | http://www.ebi.ac.uk/research/cgg/tribe/ |
| Remote Documentation | http://micans.org/mcl/man/mcx.html |

**mcx** can be used both from the command line and interactively, and supports
a rich set of operations such as transposition, scaling, column scaling,
multiplication, Hadamard powers and products, et cetera.
